# Species Dependence of SYTO 9 Staining of Bacteria

**DOI:** 10.3389/fmicb.2020.545419

**Published:** 2020-09-03

**Authors:** Cushla McGoverin, Julia Robertson, Yaqub Jonmohamadi, Simon Swift, Frédérique Vanholsbeeck

**Affiliations:** ^1^Department of Physics, University of Auckland, Auckland, New Zealand; ^2^The Dodd-Walls Centre for Photonic and Quantum Technologies, Auckland, New Zealand; ^3^Department of Molecular Medicine and Pathology, University of Auckland, Auckland, New Zealand

**Keywords:** enumeration, fluorescence, SYTO 9, Bacillus cereus, Escherichia coli, Salmonella enterica, Staphylococcus aureus

## Abstract

SYTO 9 is a fluorescent nucleic acid stain that is widely used in microbiology, particularly for fluorescence microscopy and flow cytometry analyzes. Fluorimetry-based analysis, i.e., analysis of fluorescence intensity from a bulk sample measurement, is more cost effective, rapid and accessible than microscopy or flow cytometry but requires application-specific calibration. Here we show the relevance of SYTO 9 for food safety analysis. We stained four bacterial species of relevance to food safety (*Bacillus cereus, Escherichia coli, Salmonella enterica* subspecies *enterica* ser. Typhimurium, *Staphylococcus aureus*) with different concentrations of SYTO 9, with and without the presence of ethylenediaminetetraacetic acid (EDTA), for varying amounts of time, to investigate the effect of these treatment parameters on fluorescence intensity. The addition of EDTA and an increased staining duration did not significantly affect fluorescence intensity, and over the bacterial cell concentration range investigated (∼10^5^–10^8^ CFU/ml) there was no significant difference in using 0.5 or 1 μM SYTO 9. The effect of bacterial cell concentration on fluorescence intensity was species specific. At different bacterial cell concentrations, the effect of species on fluorescence intensity is different. This interaction complicates the development of a general fluorimetry-based protocol for the determination of bacterial cell concentration in a mixed bacterial suspension, as would be expected from samples taken from food safety settings.

## Introduction

Applications involving fluorescence are widespread throughout microbiology as alternatives to culture-based methods. A variety of measurement techniques are used to collect fluorescence data, predominantly fluorescence microscopy (Donoso et al., 2017; Jackson et al., 2018), flow cytometry (De Sousa Guedes and De Souza, 2018; Zahavy et al., 2018), and fluorimetry (Bianchi et al., 2004). A fluorophore is required for fluorescence analyzes. Many bacterial species are not natively fluorescent at visible wavelengths of excitation and hence, a dye is added that will bind to a component of the target bacteria to generate a fluorescent signal (Sohn et al., 2009). SYTO 9 is one such dye. SYTO 9 is a blue-excited (485 nm excitation maximum), green-fluorescent (498 nm DNA, 501 nm RNA emission maxima) nucleic acid stain that is cell permeable (ThermoFisherScientific). Most importantly, however, is that SYTO 9 exhibits a significant enhancement in quantum yield upon binding to nucleic acids. SYTO 9 consequently has been used in fluorescence microscopy (Larrosa et al., 2012; Zotta et al., 2012; Tawakoli et al., 2013; Bogachev et al., 2018), flow cytometry (Lehtinen et al., 2004; Soejima et al., 2009; Larrosa et al., 2012), and fluorimetry studies (Pascaud et al., 2009; Larrosa et al., 2012).

In fluorimetry studies, the intensity of a fluorescence signal is used to quantitatively determine the amount of fluorophore present and can be used to determine the number of bacterial cells in a sample (Roth et al., 1997; Alakomi et al., 2005; Pascaud et al., 2009; Larrosa et al., 2012). The accurate determination of bacterial numbers requires a good understanding of the fluorophore and staining conditions, particularly if the aim is to analyze samples from a range of applications and conditions. Development of a general protocol for the SYTO 9 fluorescence-based enumeration of a bacterial sample, species unknown and potentially mixed, first requires a thorough analysis of the staining conditions and subsequent fluorescence signal of SYTO 9 applied to different bacterial species. Previous reports suggest there is a difference between the SYTO 9 staining of Gram-positive and Gram-negative bacterial cells (Invitrogen, 2004; Stiefel et al., 2015). Gram-positive and -negative bacteria are differentiated largely on the basis of cell envelope structure; Gram-negative bacterial cells have a peptidoglycan layer sandwiched between an outer and inner membrane, while Gram-positive bacteria have a thick peptidoglycan layer bordered by an inner membrane (Benson, 1998). A difference in SYTO 9 permeability between Gram-positive and -negative cells was shown by analysing the SYTO 9 fluorescence signal obtained from live and dead *Pseudomonas aeruginosa* and *Staphylococcus aureus.* Isopropanol treatment of bacteria was shown to kill bacterial cells while maintaining cell structural integrity. For *S. aureus*, the intensity of fluorescence varied little whether the bacterial cells were alive or dead, whereas, for *P. aeruginosa*, an 18-fold enhancement in fluorescence intensity was observed for dead cells, indicating greater permeation of the cell wall (Stiefel et al., 2015). The “most plausible explanation” (Stiefel et al., 2015) given for this observation was that SYTO 9 does not readily permeate the two cell membranes of Gram-negative bacteria; isopropanol treatment disrupts the integrity of the Gram-negative cell membranes and allows more SYTO 9 into the cell. Further, ethylenediaminetetraacetic acid (EDTA) is often used in the application of thiazole orange to Gram-negative bacteria to aid transport of the dye through the cell wall (Biosciences; Mchugh and Tucker, 2007). The application of EDTA increases the permeability of Gram-negative cells by acting on the cell surface while having little effect on viability (Leive, 1965). Treatment with EDTA causes the release of lipopolysaccharide from the cell envelope by chelating the divalent cations that stabilize the outer membrane of Gram-negative cells (Marvin et al., 1989).

A general fluorimetry protocol for the enumeration of unknown bacterial suspensions would have wide application in many fields. Here we investigated the utility of SYTO 9 as a dye for bacterial enumeration by staining *Bacillus cereus, S. aureus, Escherichia coli*, and *Salmonella enterica* subspecies *enterica* ser. Typhimurium under varying conditions (dye concentration, time of staining, and presence/absence of EDTA). The four bacterial species investigated were selected for their relevance to food safety and commonality. *E. coli* and *S.* Typhimurium are both Gram-negative bacteria, and *S. aureus* and *B. cereus* are Gram-positive bacteria. SYTO 9 binds to nucleic acid, meaning that the amount of nucleic acid in a cell influences the amount of SYTO 9 that will bind and subsequently the fluorescence intensity. Bacterial nucleic acid content was considered with respect to observed differences in SYTO 9 fluorescence. The bacterial chromosomes of *B. cereus, S. aureus, E. coli*, and *S.* Typhimurium are approximately 5.3 Mbp (Lee et al., 2015), 2.8 Mbp (Baba et al., 2008; Makarova et al., 2017), 5.1 Mbp (Minogue et al., 2014), and 4.8 Mbp (Mcclelland et al., 2001), and the guanine-cytosine (G+C) content is 35, 32, 50, and 51%, respectively. The G+C content was examined in addition to cell number and nucleic acid content, to determine if base pair preferential binding would explain any observed differences between staining conditions. To emulate potential applications, we used a dip probe and fiber optic-based fluorimeter to collect fluorescence data, showing the utility of using a relatively cost effective, robust fluorimeter for the collection of fluorescence data from bacterial suspensions.

## Materials and Methods

### Materials

*E. coli* American Type Culture Collection (ATCC) 25922, *S.* Typhimurium ATCC 14028, *S. aureus* ATCC 6538, and *B. cereus* ATCC 10702 were obtained from Cryosite Ltd. (Granville, NSW, Australia). Difco tryptic soy broth (TSB), Difco granulated agar, and sterile peptone water (0.1% w/w peptone, 0.85% w/w sodium chloride, pH 7) were purchased from Fort Richard, Auckland, New Zealand. Ethylenediaminetetraacetic acid disodium salt dehydrate (EDTA) was purchased from Sigma Aldrich, New Zealand, and SYTO 9 (3.34 mM in DMSO; catalog number S34854) was purchased from Life Technologies, Auckland, New Zealand.

### Sample Preparation

Bacterial samples were prepared using a broth culture that had been grown overnight in TSB at 37°C while being aerated with orbital shaking at 200 rpm. Twenty-fold dilutions of these broth cultures were subsequently prepared in fresh TSB; this habituation was allowed to continue until the suspension had an optical density of approximately 0.5 at 600 nm (1 cm path length), which was indicative of concentrations in the order of 10^8^ CFU/ml in the mid-exponential phase of growth. The time required was 45–60 min for all species, except *S. aureus*, which required 30–40 min. These habituated suspensions were used to produce bacterial suspensions with varying concentrations.

TSB was first removed from the sub-culture suspension by centrifugation at 4000 × *g* for 5 min and resuspending the bacterial pellet in peptone water to give the initial suspension. The initial suspension for sampling was diluted with peptone water to produce 10-, 100-, and 1000-fold diluted suspensions for sampling. These diluted suspensions were allowed to sit at room temperature for differing periods of time (0–3 h), which was dependent on the randomized order of sampling, hence each suspension was subject to plate counting, and each suspension, and the sample taken from it, could be treated as independent. Further, samples containing no bacteria, i.e., only peptone water, were also prepared.

The influence of SYTO 9 concentration, the duration of incubation with SYTO 9 prior to measurement and the presence of 1 mM EDTA on recorded fluorescence intensity was assessed. A sample was stained by aliquoting 0.9 ml of the bacterial suspension or peptone water, adding 0.05 ml of 0.02 M EDTA (EDTA present; 0.2 M tris-HCl pH 7) or 0.05 ml of 0 mM EDTA solution (EDTA absent; 0.2 M tris-HCl pH 7), and adding 0.05 ml of 0, 0.01, or 0.02 mM SYTO 9 (final concentration 0, 0.05, 1 μM SYTO 9, respectively, SYTO 9 was diluted with peptone water) and subsequently shaking the sample for the appropriate amount of time: 5, 15, 30 min. All samples were prepared in amber tubes to reduce exposure to light. At each concentration, for each bacterial species, there were three biological replicates.

### Standard Plate Count

Bacterial enumeration of samples was determined using the standard plate count method. At time point 0, a sample was taken from a non-stained bacterial suspension and serial dilutions were made using 0.85% saline. For the 1×, 10×, 100×, and 1000× diluted bacterial suspensions the 10^5^ and 10^6^, 10^5^ and 10^4^, 10^4^ and 10^3^, 10^3^ and 10^2^ dilutions, respectively, were used. These diluted samples were used to inoculate tryptic soy agar (TSA) plates (aqueous 30 g/L TSB, 15 g/L granulated agar) at time 0 of the fluorescence measurements (see section “Fluorescence Measurements” below). For each plate count a 50 μl aliquot of the sample was spread evenly onto a TSA plate and incubated at 37°C overnight. Triplicate plate counts were made for each tested sample.

### Fluorescence Measurements

Fluorescence spectra were recorded using a fiber optic dip probe instrument (Hewitt et al., 2012). The fluorimeter is fiber optic-based, developed to withstand industrial environments. The fluorimeter uses low OH silica fiber (diameter 200 μM, NA 0.22; Thorlabs Inc., Newton, NJ, United States) and a 473 nm solid-state laser (MLL-FN-473, Changchun New Industries Optoelectronics Technology Col., Ltd, China) for excitation of the sample, and the laser line is obstructed by a shutter to ensure sample illumination only during sample measurement. The laser line is split in a 2 × 2 (50:50) coupler (Thorlabs Inc., Newton, NJ, United States) to direct half of the light to a photodiode (DET36A, Thorlabs Inc., Newton, NJ, United States) for continuous power monitoring of the laser. The remaining half excites the sample, and the same probe used to excite the light is used to collect emitted light. The light passes through a longpass filter (4 mm of 495 nm and 2 mm of 515 nm longpass colored glass filters, FGL495S and FGL515S, respectively, from Thorlabs, NJ, United States) before entering the spectrometer (QE6500, Ocean Optics, DE, United States). Synchronization of instrument components and data collection occurred using a T7 LabJack data acquisition card and an in-laboratory written python script.

Each measurement was acquired using a laser with an average power of 9.2 mW (0.6 mW standard deviation) at the sample; day-to-day power fluctuations were accounted for using photodiode measurements. Each spectrum collected was the sum of three 20 ms acquisitions. The bacterial suspension was mixed using a vortex mixer before positioning the tip of the probe in the middle of the sample and taking a measurement. The fiber probe was cleaned with 70% ethanol between each measurement. Duplicate measurements were recorded. A total of 432, 431, 412, and 432 spectra were analyzed for *B. cereus, S. aureus, E. coli*, and *S. Typhimurium*, respectively. The lower number of spectra for *E. coli* was because several samples only had one spectrum, rather than duplicate spectra, recorded due to time constraints.

### Data Analysis

All data analyzes were carried out using R (version 3.6.1, R Core Team, 2019) the following packages: broman (Broman and Broman, 2019), car (Fox and Weisberg, 2019), fBasics (Wuertz et al., 2017), lubridate (Grolemund and Wickham, 2011), MESS (Ekstrøm, 2019), rhdf5 (Fischer et al., 2019), sqldf (Grothendieck, 2017), stringdist (Van Der Loo, 2014). Dark (thermal) noise was subtracted from each measurement. To account for fluctuations in power and integration timing, each spectrum was normalized to a laser power of 1 mW and 1 ms and the normalized background spectrum (peptone water) was subtracted from sample spectra. The duplicate spectra were then averaged, resulting in 216, 216, 215, and 216 averaged spectra for *B. cereus, S. aureus, E. coli*, and *S.* Typhimurium, respectively. These averaged spectra were used in subsequent statistical analyzes. The fluorescence intensity was calculated as the area below the spectrum was calculated for the spectral range 490–590 nm.

The bacterial cell concentration was converted to total number of nucleobases or number of G+C nucleobases under the assumption that an actively growing cell would have two chromosomes and a DNA:RNA ratio of 1:6 (Milo and Phillips, 2016). The number of nucleobases per cell for each species was based on the chromosome sizes and G+C contents given in the introduction.

General linear mixed (GLM) models were used to assess the influence of the different treatments and interactions thereof, on the fluorescence intensity recorded from SYTO 9 stained samples. The logarithm of integrated area, and bacterial cell concentration, or number of nucleobases or G+C nucleobases, were used in a GLM model to determine the significance of the various treatments on the measured fluorescence intensity. These GLM models only included data collected from stained samples, i.e., samples with 0.5 and 1 μM SYTO 9. The lm() function in R was used for GLM modeling and the significance of main effects and interactions was assessed using type III sum of squares. Significance was determined at a level of *p* < 0.05 for all presented analyzes. The assumption of normality of residuals was assessed by examining the Q-Q plot, box plot, and histogram of residuals. The assumption of equality of variance was assessed by examining the residuals versus fitted plot. To assess spectral variance the relative standard deviation (standard deviation divided by average and multiplied by 100) was calculated for duplicate measurements and plotted as a function of bacterial cell concentration and staining conditions.

The effect of adding EDTA, SYTO 9 concentration and the incubation duration on the fluorescence spectrum of peptone water alone were investigated using a GLM model applied to the integrated area values calculated from spectra that were only normalized to 1 mW and 1 ms, no background was subtracted. The average intensity of fluorescence when SYTO 9 alone was added to peptone water was calculated by pooling all samples containing no bacteria and stained with the respective concentration of SYTO 9.

Native fluorescence of the samples was investigated. These were samples of varying bacterial concentration to which SYTO 9 was not added. GLM models were applied to the integrated area values calculated from spectra that were only normalized to 1 mW and 1 ms and had the background subtracted.

## Results

### Peptone Water Fluorescence

**FIGURE 1:**
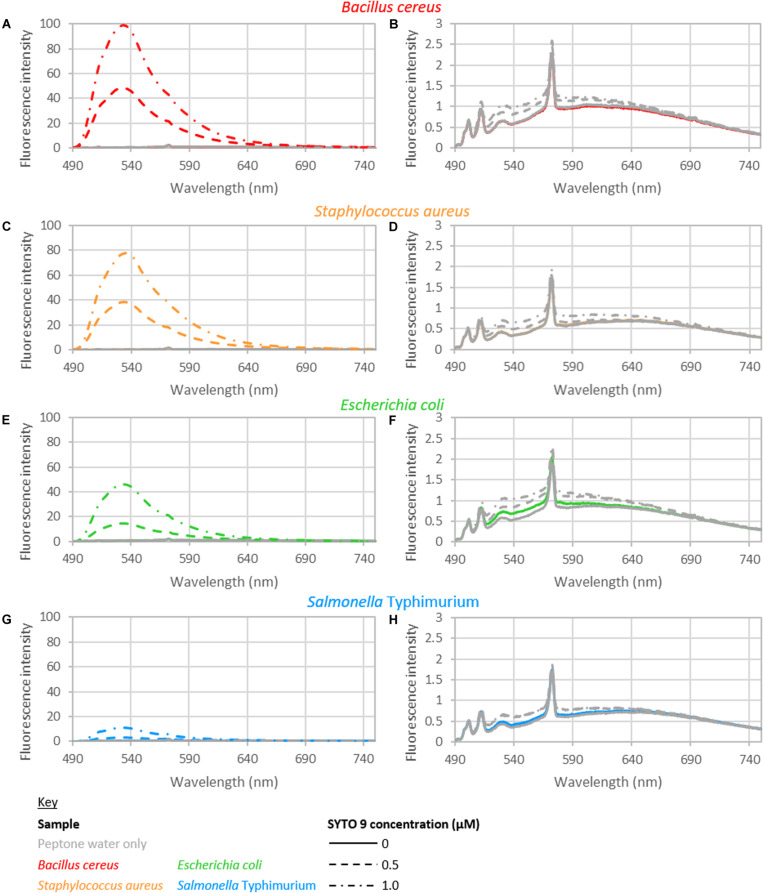
Fluorescence spectra of *B. cereus* **(A,B)**, *S. aureus* **(C,D)**, *E. coli* **(E,F)**, and *S.* Typhimurium **(G,H)**. **(B,D,F,H)** Show the blank and control spectra: bacteria with no dye (colored solid line); peptone water alone (gray solid line); 0.5 μM SYTO 9 in peptone water (gray dashed line); 1.0 μM SYTO 9 in peptone water (gray dotted-dashed line). The spectra shown are recorded from bacterial suspensions with concentrations of approximately 10^8^ CFU/ml bacteria (*B. cereus* 1.46 × 10^8^; *S. aureus* 3.49 × 10^8^; *E. coli* 3.50 × 10^8^; *S.* Typhimurium 2.46 × 10^8^ CFU/ml). The staining treatment used for these samples was 0 mM EDTA and 15 min staining. Each spectrum shown is the average of duplicate spectra recorded with the specified conditions, and normalized to 1 ms and 1 mW. The peptone water spectrum served as the background spectrum in data analyzes. The background spectrum was not subtracted from the spectra presented here to highlight the minimal difference of the control spectra to blank spectrum.

The intensity from peptone water did not vary significantly with respect to the addition of EDTA or the time held for incubation. The fluorescence intensity did vary significantly with the concentration of SYTO 9 dye. However, only a small increase in fluorescence was observed with the addition of SYTO 9 to peptone water (Figure 1). The average intensity of fluorescence observed across 490–590 nm, from samples with no bacteria was 1.75, 1.85, and 1.92 for samples stained with 0, 0.5, and 1 μM SYTO 9, respectively.

### Unstained Bacterial Suspensions

The spectra of the unstained suspensions were very similar to peptone water spectra (Figure 1). The GLM indicated there was a significant interaction between the bacterial cell concentration and species when considering fluorescence intensity in terms of bacterial cell concentration. No other interactions or main effects were significant. When examining total nucleobase content, the only significant interaction was between total nucleobase content and species; no main effects were observed. No interactions or main effects were significant when considering G+C nucleobase content.

### Stained Bacterial Suspensions

#### Species

**FIGURE 2:**
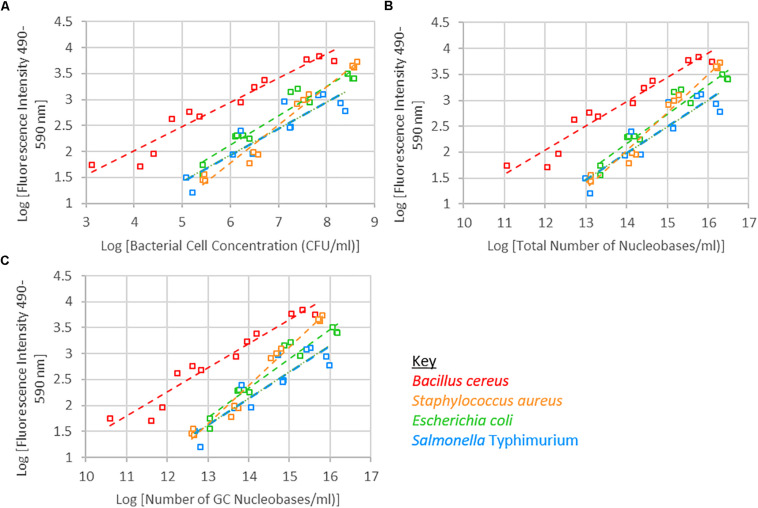
Logarithm of fluorescence intensity (490–590 nm) against logarithm of bacterial cell concentration (CFU/ml) **(A)**, total number of nucleobases **(B)** or number of GC nucleobases **(C)** for samples treated with 1 μM SYTO 9, incubated for 15 min and 0 mM EDTA are shown.

A significant interaction was observed between species and bacterial cell concentration. Of the four species of bacteria investigated, the fluorescence intensity of *B. cereus* tended to be higher than the other three species. S. Typhimurium tended to have a lower fluorescence intensity than the other species, while *S.* Typhimurium and *E. coli* exhibited similar fluorescence intensities across the bacterial cell concentration range investigated (Figure 2 and Supplementary Figure 1). When examining fluorescence intensity with respect to total nucleobase content, a significant interaction between species and total nucleobase content was observed. Similarly, when examining G+C nucleobase content a significant species and G+C nucleobase content interactions was observed.

#### Dye Concentration

**FIGURE 3:**
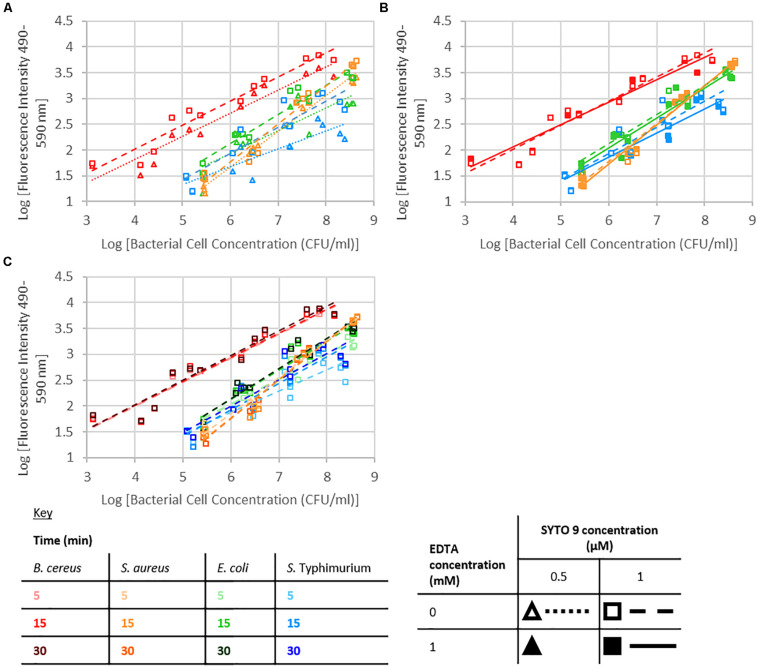
Logarithm of fluorescence intensity (490–590 nm) against logarithm of bacterial cell concentration (CFU/ml) for *B. cereus, S. aureus*, *E. coli*, and *S.* Typhimurium and treated with: **(A)** 0 mM EDTA, stained for 15 min, **(B)** 1 μM SYTO 9, stained for 15 min, **(C)** 1 μM SYTO 9 and 0 mM EDTA.

Fluorescence from the SYTO 9 fluorophore is dramatically enhanced upon binding to nucleic acids (Figure 2; Thermofisherscientific, 2019). A significant interaction between dye concentration and bacterial cell concentration, or nucleobase content or G+C content was not observed. SYTO 9 concentration was not a significant effect; however, at the highest investigated bacterial cell concentrations there was an observable difference between fluorescence intensity when 0.5 or 1 μM SYTO 9 was used (Figure 3).

#### EDTA Concentration

The presence of 1 mM EDTA had no significant effect on the intensity of fluorescence, and no significant interactions with EDTA were observed (Figure 3).

#### Staining Duration

Staining duration did not have a significant effect on fluorescence intensity. For the investigated Gram-positive species, there were no obvious differences in the data collected using different staining durations; however, in the case of the Gram-negative species investigated, there was a trend for the 5 min staining to lead to a lower fluorescence signal than 15 or 30 min staining (Figure 3).

#### Repeatability

**FIGURE 4:**
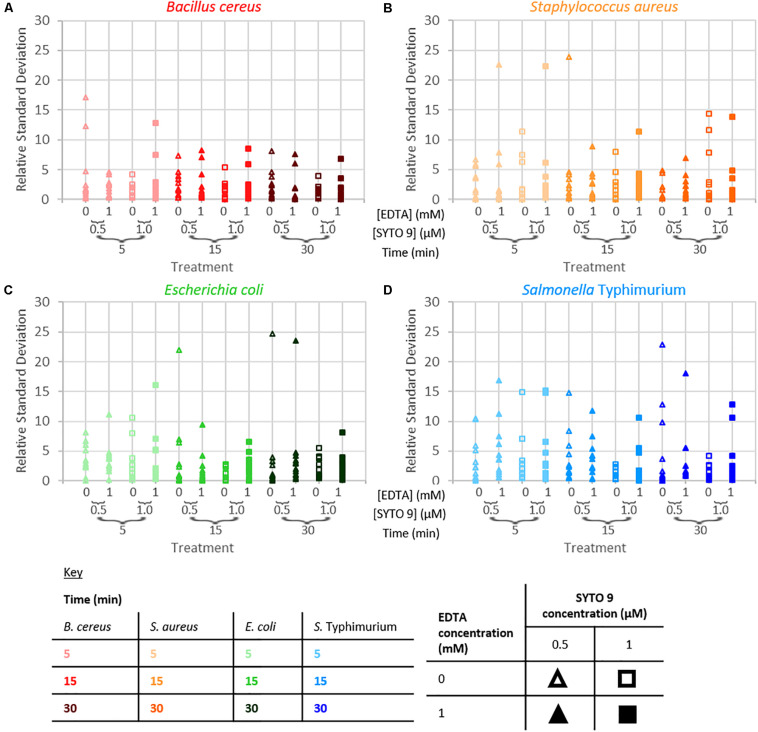
Relative standard deviation of logarithm of fluorescence intensity (490–590 nm) for samples as a function of staining treatment for *B. cereus* **(A)**, *S. aureus* **(B)**, *E. coli* **(C),** and *S.* Typhimurium **(D)**.

**FIGURE 5:**
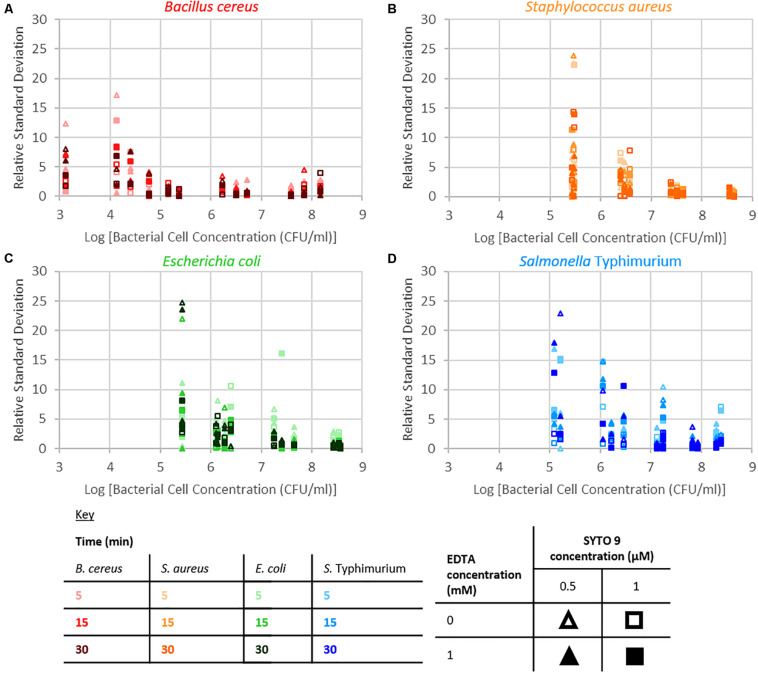
Relative standard deviation of logarithm of fluorescence intensity (490–590 nm) for samples against the logarithm of bacterial cell concentration for *B. cereus* **(A)**, *S. aureus* **(B)**, *E. coli* **(C)**, and *S.* Typhimurium **(D)** for the different treatment conditions as defined by the key.

The repeatability of the collected fluorescence intensity (490–590 nm) was assessed by calculating the residual standard deviation of the two measurements taken from each sample. Examination of relative standard deviation as a function of staining treatment indicated that no one treatment leads to exceptionally better repeatability than the others (Figure 4). Though the staining condition of 15 min incubation duration and 1 μM SYTO 9 with no EDTA was generally most repeatable across the four species. Measurement repeatability (lower relative standard deviation indicates a more repeatable measurement) dramatically reduced as the concentration of bacteria decreased (Figure 5). The concentration-based reduction in repeatability was smallest for *B. cereus*.

## Discussion

The fluorescence signal from this SYTO 9-nucleic acid complex in bacterial suspensions was examined to determine if and how staining time, the addition of EDTA, the dye concentration, and the species of bacteria affect the intensity of this signal. The intensity of fluorescence became more variable as bacterial concentration decreased. The bacterial samples are suspensions of particulates (cells) in peptone water solutions; detection of fluorescence from these cells requires the cell to be in the excitation/collection volume. The probability of bacterial cells being in this sample volume is a function of both bacterial cell concentration and cell size; the higher the bacterial cell concentration or the larger the cell size, the higher the probability the cell will be within the sampling volume. *B. cereus* cells are the largest (1 × 3–5 μm rods) of the investigated species (Harris et al., 2002; Reshes et al., 2008; Senesi and Ghelardi, 2010; Fabrega and Vila, 2013), consequently we speculate that this is the reason why the fluorescence signal at low bacterial concentrations from *B. cereus* was least variable of the species investigated.

The bacterial cell concentration-species interaction significantly affected fluorescence intensity. At the same cell concentrations, the different species of bacteria exhibited different levels of fluorescence. The nucleic acid content within cells of each of these species differ, which may account for the significant interaction observed between bacterial cell concentration and species with respect to fluorescence intensity. Accounting for differences in nucleic acid content, and specifically G+C nucleic acid content, did not remove the significant interaction (Figure 3). The observed interaction may be a consequence of cell permeability differences between Gram-positive and Gram-negative bacteria.

An increase in fluorescence intensity is expected with increasing SYTO 9 concentration as more SYTO 9 will bind the nucleic acids present. This increase will be observed until the point that the stain to nucleic acid ratio is high enough that the presence of further SYTO 9 molecules quench fluorescence. A significant interaction between dye concentration and bacterial cell concentration, or nucleobase content or G+C content was not observed. The difference in fluorescence intensity between when 0.5 or 1 μM SYTO 9 was used was obvious when the number of bacterial cells was high. However, as the bacterial numbers decreased this difference was less obvious. There are two potential reasons for this; at the high bacterial concentrations 0.5 μM SYTO 9 is not concentrated enough to occupy all nucleic acid binding sites; alternatively, this is potentially a consequence of SYTO 9 self-quenching at lower bacterial cell concentrations.

Neither the presence of 1 mM EDTA nor the amount of time between the addition of stain and the recording of fluorescence had a significant effect on the resulting fluorescence intensity. EDTA was added to the bacterial samples as it has been used with thiazole orange to improve staining of Gram-negative cells. The concentration of 1 mM EDTA was selected for use on the basis of previous publications (Biosciences; Mason et al., 1998; Mchugh and Tucker, 2007).

For greatest accuracy, enumeration of bacteria based on the fluorescence intensity of SYTO 9 is best used in applications where monocultures of bacteria are targeted (e.g., fermentations in the food industry). This mitigates the confounding factor of species-specific levels of fluorescence. Any application of fluorimetry-based enumeration of bacteria will require sample preparation in the form of a separation step to isolate the bacteria and in many cases a concentration step to improve measurement repeatability. A variety of methods for separation (e.g., filtration, microfluidics) and concentration (e.g., magnetophoresis, microfluidics) have been previously published, and the incorporation of these methods with fluorimetry-based enumeration will be the focus of future work (Brehm-Stecher et al., 2009; Jung et al., 2020; Kim and Oh, 2020).

## Conclusion

Real-world food samples have complex microbiomes comprised of a number of different bacterial types, with different physiological histories (e.g., age, exposure to stressors) and heterogeneity of cell size and shape. An all-purpose fluorescence intensity calibration for accurate enumeration of bacteria is not possible. This is because there is a significant interaction between species and bacterial concentration, i.e., all bacterial species are not equal in terms of SYTO 9 based fluorescence of a sample. Beyond this intensity effect, we speculate that spectral repeatability is affected by the size of bacterial cells. Suspension samples taken from industrial food safety settings are likely to be a mixture of unidentified bacterial species. Hence, development of a general protocol for fluorescence intensity-based enumeration of industrial suspension samples will have analytical challenges. Enumeration to an order of magnitude may be the best possible result but would still have real-world application in the bacterial analyzes of food. This fluorescence-based approach to enumeration could be better applied to applications where monocultures are used, for example, fermentations in the food, ethanol, pharmaceutical, and biotechnology industries. In terms of obtaining a reliable fluorescence signal from an axenic population of bacteria, the staining conditions of 15 min incubation duration and 1 μM SYTO 9 with no EDTA present will give reasonable results in terms of signal intensity and repeatability across the bacterial concentration range of 10^5^–10^8^ CFU/ml.

## Data Availability Statement

The raw data supporting the conclusions of this article will be made available by the authors, without undue reservation, to any qualified researcher.

## Author Contributions

CM conceived, designed, and performed experiments, analyzed data, prepared the manuscript, and approved the final draft. JR performed experiments, reviewed drafts of the manuscript, and approved the final draft. YJ provided computer code for operation for the fluorimeter and approved the final draft. FV and SS were involved in project administration, reviewed drafts of the manuscript, and approved the final draft. All authors contributed to the article and approved the submitted version.

## Conflict of Interest

The authors declare that the research was conducted in the absence of any commercial or financial relationships that could be construed as a potential conflict of interest.
