# Supplementary material for: Species Dependence of SYTO 9 Staining of Bacteria
Source: Front Microbiol. 2020 Sep 3;11:545419. doi: 10.3389/fmicb.2020.545419 (PMC7494787; doi:10.3389/fmicb.2020.545419)
Supplement: Supplementary file 2 [file Table_1.DOCX]

Supplementary Material


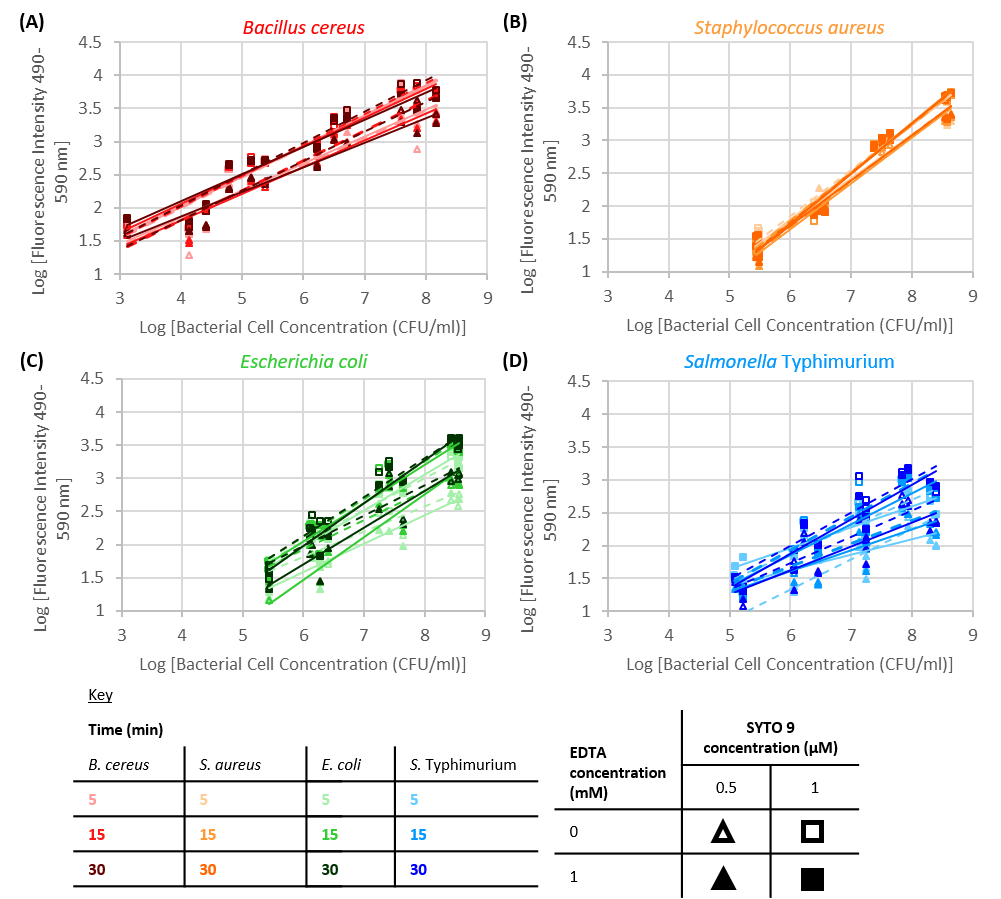


**Supplementary Figure 1.** Logarithm of fluorescence intensity (490-590 nm) against logarithm of bacterial cell concentration (CFU/ml) of *B. cereus* (A), *S. aureus* (B), *E. coli* (C) and *S.* Typhimurium (D) for the different treatment conditions as defined by the key.
